# Supplementary material for: Risk factors, ethnicity and dementia: A UK Biobank prospective cohort study of White, South Asian and Black participants
Source: PLoS One. 2022 Oct 12;17(10):e0275309. doi: 10.1371/journal.pone.0275309 (PMC9555673; doi:10.1371/journal.pone.0275309)
Supplement: S1 File — (DOCX) [file pone.0275309.s001.docx]

# Supplementary material

## Risk factors of interest

We searched Medline, Embase and Allied and Complementary Medicine databases from inception until 25^th^ September 2020 for systematic reviews of dementia risk factors published using search terms “review” OR “meta-analysis” AND “risk factors” AND “dementia” OR “AD”. We set no limits on language. We included systematic reviews that considered multiple potentially modifiable dementia risk factors across the life course where authors had included multiple cohort studies for each risk factor and weighed up the evidence for each risk factor. We excluded reviews only focused on one risk factor or one category such as, for example, environmental factors or dietary factors. Our search retrieved 2355 results, of which the majority were excluded because they were not examining risk factors for dementia. We considered 13 full-text papers but excluded: one because it was focused on the additive effect of having multiple risk factors^1^; one as it was not a systematic review^2^; one as it was only focused on early life risk factors^3^; one as it only considered environmental toxins^4^; and one as it only focused on midlife risk factors^5^. We included eight systematic reviews^6-13^ from our search and additionally identified the Lancet Commissions on dementia as relevant for this topic^14,15^.

We considered risk factors relevant if there was good quality evidence from prospective cohort studies showing an association between a risk factor and incident dementia and they were potentially modifiable. We selected risk factors with a meta-analysis studying a narrowly specified risk factor (not “hormones” for example); incorporating studies examining risk factors preceding dementia outcome by ≥10 years to reduce the risk of reverse causality; with some indication about which stage of life the risk factors were relevant in, i.e. early, mid or later life; and where specified clinical criteria were used to define risk factors. We excluded risk factors that could be part of the diagnosis of dementia such as motor symptoms related to Parkinsons disease or stroke.

The Lancet Commission on dementia prevention, intervention and care^15^ presented evidence for nine potentially modifiable risk factors (low education, hearing loss, midlife hypertension and obesity, depression, social isolation, diabetes, smoking, physical inactivity). The Lancet Standing Commission on dementia ^14^ presents 12 risk factors over the life course which are consistently associated with all-cause dementia: low education, head injury, hearing loss, midlife hypertension and obesity, depression, social isolation, excessive alcohol use, diabetes, smoking, physical inactivity and air pollution. For depression and diabetes there is no clear evidence as to whether treatment with medication mitigates the risk of subsequent dementia. The other systematic reviews we included provided evidence for further risk factors. We then searched in the UK Biobank data showcase to see if each risk factor or a suitable proxy was measured in participants.

| **Risk factor** | **UK Biobank** |
| --- | --- |
| Less education | ✓ |
| Excess alcohol consumption | ✓ |
| Physical inactivity | ✓ |
| Sleep disorders | × |
| Smoking | ✓ |
| High total cholesterol | ✓ |
| Depression | ✓ |
| Diabetes | ✓ |
| Hearing loss | ✓ |
| Hypertension | ✓ |
| Traumatic brain injury* | × |
| Social isolation | ✓ |
| Anti-hypertensives (protective) | ✓ |
| Obesity | ✓ |
| Air pollution | ✓ |

*too large or complexly formatted to be distributed as part of a standard phenotype database

Excluded factors with reasons (all from Anstey et al except those marked with * which are from Di Marco et al)

| **Risk factor** | **Reason for exclusion** |
| --- | --- |
| Bilingualism | Unclear criteria used to define it |
| Cognitive engagement | Varied measures, unclear definitions |
| Diet | Varied subcategories, unclear classification |
| Social engagement | Varied measures, no clear definition |
| Stress | No clear definition |
| Arthritis | Only two cohort studies, both only 5 years follow up |
| Atrial fibrillation | Known cause of cerebrovascular events |
| Anxiety | <10 years follow up |
| Cancer | Varied clinical conditions, variation in determining exposure |
| Carotid atherosclerosis | Known cause of cerebrovascular events |
| Homocysteine | Only one study across both systematic reviews followed up (only women) for more than 10 years. Rest were shorter follow up or case-control studies |
| Thyrotropin | Only one study with follow up longer than 10 years |
| Testosterone | Only one study with follow up longer than 10 years |
| Inflammatory markers | Unclear clinical criteria |
| Metabolic syndrome | <10 years follow up in all included studies |
| Motor function | Unclear clinical criteria |
| NSAIDs | Mixed evidence – different medications, different durations |
| Peripheral artery disease | <10 years follow up in all included studies |
| Renal disease | <10 years follow up in all included studies |
| Serum uric acid | Unclear clinical criteria |
| Stroke | Cerebrovascular event part of diagnostic criteria for vascular dementia |
| Antacids | Unclear clinical criteria |
| Benzodiazepines | Unclear length of follow-up |
| Insulin sensitisers | Unclear clinical criteria |
| Coffee and tea consumption | Varied measures, contradictory evidence |
| Pesticides | <10 years follow up |
| Statins | Only one cohort study with >10 years follow up |

We also did not include antihypertensives as a separate protective factor as they were used to determine if participants had a history of hypertension.

## Unadjusted estimates

| **Risk factor** | **White** | | | | **South Asian** | | | | **Black** | | | |
| --- | --- | --- | --- | --- | --- | --- | --- | --- | --- | --- | --- | --- |
|  | **HR** | | **95% CI** | | **HR** | | **95% CI** | | **HR** | | **95% CI** | |
|  |  | Lower | | Upper |  | Lower | | Upper |  | Lower | | Upper |
| **Education (above GCSE vs below)** | 0.63 | 0.60 | | 0.67 | 0.88 | 0.52 | | 1.49 | 0.74 | 0.48 | | 1.14 |
| **Hypertension** | 1.67 | 1.58 | | 1.75 | 2.75 | 1.69 | | 4.47 | 1.66 | 1.07 | | 2.60 |
| **Hearing loss** | 1.42 | 1.35 | | 1.50 | 1.44 | 0.92 | | 2.24 | 1.53 | 1.01 | | 2.32 |
| **Obesity** | 1.17 | 1.11 | | 1.24 | 0.90 | 0.54 | | 1.51 | 0.97 | 0.64 | | 1.47 |
| **Drinking >21 units alcohol per week vs drinking 1-21 units** | 1.04 | 0.97 | | 1.12 | 2.82 | 1.31 | | 3.32 | 1.64 | 0.69 | | 3.88 |
| **Current smoker** | 1.20 | 1.10 | | 1.31 | 0.98 | 0.40 | | 2.43 | 0.86 | 0.38 | | 1.98 |
| **History of depression vs no reported history** | 1.19 | 1.13 | | 1.26 | 2.09 | 0.51 | | 3.14 | 0.93 | 0.55 | | 1.58 |
| **Less than daily/almost daily social contact vs less than this** | 1.30 | 1.22 | | 1.38 | 1.67 | 0.92 | | 3.03 | 1.34 | 0.88 | | 2.05 |
| **Physical inactivity (<WHO recommendations)** | 1.60 | 1.49 | | 1.72 | 1.49 | 0.90 | | 2.45 | 1.28 | 0.78 | | 2.10 |
| **PM_2.5_ Air pollution >WHO recommendation** | 1.18 | 1.11 | | 1.24 | 1.54 | 0.95 | | 2.50 | 1.13 | 0.68 | | 1.85 |
| **Diabetes** | 2.55 | 2.37 | | 2.75 | 2.78 | 1.78 | | 4.37 | 2.15 | 1.38 | | 3.34 |
| **High total cholesterol (>6.5mmol/l)** | 0.75 | 0.71 | | 0.80 | 0.43 | 0.16 | | 1.17 | 0.97 | 0.53 | | 1.79 |

1. Peters R, Booth A, Rockwood K, Peters J, D’Este C, Anstey KJ. Combining modifiable risk factors and risk of dementia: a systematic review and meta-analysis. *BMJ open* 2019; **9**(1): e022846.

2. Menge DMa, Nair N, Vijaya Kumar PRA. Preventing Dementia: Shifting Focus Towards Potential Modifiable Risk Factors. *Journal of Young Pharmacists* 2019; **11**(3).

3. Wang X-J, Xu W, Li J-Q, Cao X-P, Tan L, Yu J-T. Early-Life risk factors for dementia and cognitive impairment in later life: a systematic review and Meta-Analysis. *Journal of Alzheimer's Disease* 2019; **67**(1): 221-9.

4. Killin LO, Starr JM, Shiue IJ, Russ TC. Environmental risk factors for dementia: a systematic review. *BMC geriatrics* 2016; **16**(1): 1-28.

5. Li X-Y, Zhang M, Xu W, et al. Midlife Modifiable Risk Factors for Dementia: A Systematic Review and Meta-analysis of 34 Prospective Cohort Studies. *Current Alzheimer Research* 2019; **16**(14): 1254-68.

6. Liang J-h, Lu L, Li J-y, et al. Contributions of Modifiable Risk Factors to Dementia Incidence: A Bayesian Network Analysis. *Journal of the American Medical Directors Association* 2020.

7. Anstey KJ, Ee N, Eramudugolla R, Jagger C, Peters R. A systematic review of meta-analyses that evaluate risk factors for dementia to evaluate the quantity, quality, and global representativeness of evidence. *Journal of Alzheimer's Disease* 2019; (Preprint): 1-21.

8. Bellou V, Belbasis L, Tzoulaki I, Middleton LT, Ioannidis JP, Evangelou E. Systematic evaluation of the associations between environmental risk factors and dementia: An umbrella review of systematic reviews and meta-analyses. *Alzheimer's & Dementia* 2017; **13**(4): 406-18.

9. Hazar N, Seddigh L, Rampisheh Z, Nojomi M. Population attributable fraction of modifiable risk factors for Alzheimer disease: A systematic review of systematic reviews. *Iranian journal of neurology* 2016; **15**(3): 164.

10. Xu W, Tan L, Wang H-F, et al. Meta-analysis of modifiable risk factors for Alzheimer's disease. *Journal of Neurology, Neurosurgery & Psychiatry* 2015; **86**(12): 1299-306.

11. Hersi M, Irvine B, Gupta P, Gomes J, Birkett N, Krewski D. Risk factors associated with the onset and progression of Alzheimer’s disease: A systematic review of the evidence. *Neurotoxicology* 2017; **61**: 143-87.

12. Deckers K, van Boxtel MP, Schiepers OJ, et al. Target risk factors for dementia prevention: a systematic review and Delphi consensus study on the evidence from observational studies. *International journal of geriatric psychiatry* 2015; **30**(3): 234-46.

13. Di Marco LY, Marzo A, Munoz-Ruiz M, et al. Modifiable lifestyle factors in dementia: a systematic review of longitudinal observational cohort studies. *Journal of Alzheimer's disease* 2014; **42**(1): 119-35.

14. Livingston G, Huntley J, Sommerlad A, et al. Dementia prevention, intervention, and care: 2020 report of the Lancet Commission. *The Lancet* 2020.

15. Livingston G, Sommerlad A, Orgeta V, et al. Dementia prevention, intervention, and care. *The Lancet* 2017; **390**(10113): 2673-734.
