# Supplementary material for: Risk factors, ethnicity and dementia: A UK Biobank prospective cohort study of White, South Asian and Black participants
Source: PLoS One. 2022 Oct 12;17(10):e0275309. doi: 10.1371/journal.pone.0275309 (PMC9555673; doi:10.1371/journal.pone.0275309)
Supplement: Attachment — Submitted filename: Review_D-21-24344.pdf [file pone.0275309.s002.pdf]

In this paper, authors have leveraged the rich UK Biobank data to parse out similarity and dissimilarity, if any, of risk factors for dementia among different ethnic populations of the UK. The paper is extremely well written using reasonable statistical methods.
I have following minor comments/suggestions for the authors to consider:

1. The paper is heavy at the data side and therefore, a pictorial presentation of their cohort diversity and risk factors could aid in readability. Authors could consider pie charts to show the diversity (number/age/sex etc) and characteristic of their samples.
2. The two ethnic minorities, Black and South Asians, are certainly underpowered (less than 2 % of the total subject) as rightly pointed out by the authors. Wondering if resampling the White population in this study using bootstrapping would still lead to similar statistical outcome or there is heterogeneity in the White population that is getting masked by oversampling?
3. Can the authors also calculate and compare the effect size of the contribution of the risk factors leading to dementia in the three ethnic populations?
4. If I am not wrong, UK Biobank does collect family history that can be helpful to know if the parents or grandparents suffered from dementia. Wondering if this information is available for the studied cohort? If yes, it will be good to factor it in and see if it could be considered as a predictive factor.
5. The main results of the paper are all buried in text. A correlation matrix using heat maps or similar visual representations would be very helpful.
6. In table 1, row 3, 'Hypertension', was confirmed by either self-report or medication history. In the discussion section, author's have mentioned the following '...depression, hearing, hypertension and diabetes were all self-reported and therefore may not be accurate'. This is confusing. If medication records are available, why not limit the observation to those subjects excluding the self-diagnosed/self-reported cases?
7. In the 'Analysis of association of risk factors with dementia-Ethnicity' section, the term 'Asian Bangladeshi' is confusing. What is the difference between Asain Bangladeshi and Bangladeshi?
8. In this study, participants aged 55 and over were chosen with a follow-up period of 15 years. So the age range of the sample is 55-70 years. It seems that the sample could be further characterized into early- (subjects with age below 65 years) and late-onset dementia. The difference between the risk factors between these two types should be clinically relevant, elevating the impact of the paper.
