# Supplementary material for: Risk factors, ethnicity and dementia: A UK Biobank prospective cohort study of White, South Asian and Black participants
Source: PLoS One. 2022 Oct 12;17(10):e0275309. doi: 10.1371/journal.pone.0275309 (PMC9555673; doi:10.1371/journal.pone.0275309)
Supplement: Attachment — Submitted filename: Response to reviewers.docx [file pone.0275309.s003.docx]

**Response to reviewers**

In this paper, authors have leveraged the rich UK Biobank data to parse out similarity and

dissimilarity, if any, of risk factors for dementia among different ethnic populations of the UK.

The paper is extremely well written using reasonable statistical methods.

- Thank you for your positive comments

I have following minor comments/suggestions for the authors to consider:

1. The paper is heavy at the data side and therefore, a pictorial presentation of their

cohort diversity and risk factors could aid in readability. Authors could consider pie

charts to show the diversity (number/age/sex etc) and characteristic of their samples.

- We considered pictorial representation but concluded that as there are three main ethnic groups and these are manageable within a table, that this would be easily understood and assimilated by readers.

2. The two ethnic minorities, Black and South Asians, are certainly underpowered (less

than 2 % of the total subject) as rightly pointed out by the authors. Wondering if

resampling the White population in this study using bootstrapping would still lead to

similar statistical outcome or there is heterogeneity in the White population that is

getting masked by oversampling?

- Although the percentage of people from minority ethnic groups was relatively small, total number was over 2700 for both groups. Ethnicity data was relatively complete so we did not think it appropriate to use statistical techniques to make assumptions about ethnicity as this could bias the results.

3. Can the authors also calculate and compare the effect size of the contribution of the

risk factors leading to dementia in the three ethnic populations?

- This is a good suggestion but was not part of the aims of this paper.

4. If I am not wrong, UK Biobank does collect family history that can be helpful to know

if the parents or grandparents suffered from dementia. Wondering if this information

is available for the studied cohort? If yes, it will be good to factor it in and see if it

could be considered as a predictive factor.

- Yes UK Biobank does collect family history but as this is not a potentially modifiable risk factor we did not include this in our analyses.

5. The main results of the paper are all buried in text. A correlation matrix using heat

maps or similar visual representations would be very helpful.

- We are not sure how a correlation matrix could be used to illustrate our findings. We have used tables wherever possible, to avoid over-writing the results.

6. In table 1, row 3, ‘Hypertension’, was confirmed by either self-report or medication

history. In the discussion section, author’s have mentioned the following

‘...depression, hearing, hypertension and diabetes were all self-reported and

therefore may not be accurate’. This is confusing. If medication records are available,

why not limit the observation to those subjects excluding the

self-diagnosed/self-reported cases?

- We agree this is misleading. We have now revised it to say that hypertension was either through self-reported diagnosis or reported use of hypertensive medications. As both of these are self-report measures and people are more likely to under-report than over-report their medical conditions, we defined people with hypertension as those who reported either or both.

7. In the ‘Analysis of association of risk factors with dementia-Ethnicity’ section, the

term ‘Asian Bangladeshi’ is confusing. What is the difference between Asain

Bangladeshi and Bangladeshi?

- We have now removed the word “Asian” from this line so it now reads “South Asian (Bangladeshi, Indian, Pakistani)”

8. In this study, participants aged 55 and over were chosen with a follow-up period of 15

years. So the age range of the sample is 55-70 years. It seems that the sample could

be further characterized into early- (subjects with age below 65 years) and late-onset

dementia. The difference between the risk factors between these two types should

be clinically relevant, elevating the impact of the paper.

- We considered doing this but the majority of cases are late onset and if we separate cases into late and early onset then stratifying by ethnic group results in very small sample sizes for the analyses.
