# Supplementary material for: Risk factors, ethnicity and dementia: A UK Biobank prospective cohort study of White, South Asian and Black participants
Source: PLoS One. 2022 Oct 12;17(10):e0275309. doi: 10.1371/journal.pone.0275309 (PMC9555673; doi:10.1371/journal.pone.0275309)
Supplement: Attachment — Submitted filename: Response to reviewers_second round.docx [file pone.0275309.s004.docx]

**Response to reviewers**

Reviewer #1: (No Response)

Reviewer #2: This study was analysed and written very well. Although there are various factors about ethnicity and dementia risks left to explore, the study utilized data very well and made a tangible conclusion. In addition, a Kaplan-Meyer plot of the dementia conversion rate on the three ethics would help readers to get information more clearly. It would be beneficial to add some suggestions for future research on how to collect data that should be done to disentangle the ethnic issues on dementia. My two comments are merely soft suggestions, the author can choose to correction or not.

- Thank you for these suggestions. As the number of dementia cases in the minority ethnic groups is so low we did not think we would have sufficient data points for a Kaplan Meier plot. We have suggested in our conclusions that future studies use a larger sample of people from minority ethnic groups and a more representative population to disentangle these questions further.

Reviewer #3: The study is an interesting study that investigates the risk factors, ethnicity, and dementia. The result of APOE in these 3 subgroups of the population was also interesting. I have some comments as follows.
1. The diagnosis of dementia which was established through self-report or linked electronic health records in the UK Biobank using a defined algorithm and recognised ICD9 and ICD10 codes. I am wondering if the self-report contained high accuracy or not, especially for those in an ambulatory care or community care who had not been hospitalized or dead where the accuracy might be higher.

- We were not able to ascertain the validity of dementia diagnosis in this study but previous studies using the UK Biobank have shown a high validity for dementia diagnosis and we have cited this literature in our introduction.

2. The 2 ethnic groups were relatively small in proportion (3590 South Asian and 2766 Black people). I could understand that it would be a normal distribution of such a proportion in the general population in the UK, but the incidence of dementia and rare risk factors might be less precised and provided wide confidence interval.

- We agree the sample sizes are relatively small and have mentioned this in our limitations.

3. The risk factors might be defined at the beginning of data collection. I am not sure if the risk factors could change over time such as from current smoke to non-smoke. Would this lead to potential misclassification of the risk factors.

- We agree the risk factor classification could change over time and have now added this sentence to our discussion: “Risk factor status may have changed over the course of the study but we did not take this into account, for example, some people may have stopped smoking or people may have developed hypertension who did not have it at baseline.”

4. I am wondered if the other illnesses which might develop later after the study entry and could increase the risk of dementia were collected and analysed.

- We have addressed this point with the sentence copied above – as we only measured risks at baseline we may have missed some.
